# Medical News and Miscellany

**Published:** 1891-03

**Authors:** 


### Medical News and Miscellany.

Dr. T. J. Murph, of Nacogdoches, died February 28th, after a long illness.

Read the resolutions by the Austin District Medical Society, under Society Notes.

Dr. J. H. Burleson, of Huntsville, Texas, was married on the 20th instant, to Miss E. Woodall, of that city.

Wanted.—A few copies of the January, '91, number, and also of July, '89. Will pay for them, or give credit on subscription.

Dr. St. Cloud Cooper has returned from San Antonio to Jefferson, his former residence. He removed to San Antonio last fall.

See Dr. Hammond's new advertisement. The Doctor writes us that he has more patients from Texas than from any other State.

The spring session of the Marion Sims Medical College begun Monday, March 16, and will close May 15. They have an able faculty.

Dr. T. P. Davis, of Canton, Texas, has just returned home after a pleasant and profitable attendance upon a special course of lectures in Atlanta, Georgia.

Removals.—Dr. D. F. Kirkpatrick has removed from Fort Stockton to Argyle, Denton county.

Dr. J. W. Smith has removed from Pilot Point to Henrietta.

The Danbury Medical Publishing Company had their office (*New England Medical Journal*) burned February 28th ult. But with true pluck they went to work, and now announce that all their publications will be out nearly on time in April.

---

**To our Subscribers.**--If you want your JOURNALS bound neatly and at northern prices, send them to the JOURNAL'S printer, at Austin. Mr. John Southgate, the famous book-binder, is now with the office. Address us, or Von Boeckmann, proprietor.

---

**Married.**—In Jefferson, Texas, Monday, March 7, (inst.) by Rev. H. M. Scudder, of Marshall, Texas, Mr. Melzar H. Schluter to Miss Fannie Clopton, both of Jefferson. The bride was one of Jefferson's handsome and most popular young ladies, and a queenly figure in social circles. She is an adopted daughter of Dr. and Mrs A. G. Clopton. The groom has been a citizen of Jefferson several years, and is the senior member of the mercantile firm of Schluter & Harper.

---

**In the editorial** on "Homeopathy," etc., there are several errors of the printers, overlooked in proof reading; for instance, for "electric delegation" read "Eclectic delegation; for "medicine doses" read "medicinal doses;" for "tacily" read "tacitly;" for "easnet" read "earnest," etc. This editorial was published in pamphlet form in advance of the JOURNAL, by a committee of the physicians of Austin, on behalf of the friends of the medical bill then pending in the Senate. It was done to correct certain erroneous impressions that had been created by the industry of the opponents of the bill.

---

**The** late Dr. J. B. Raimey, of Gilmer, whose death, December 11, 1890, was announced in the last issue of the JOURNAL, was a native of Kentucky. He graduated from the Kentucky School of Medicine in June, 1882, and removed to Texas soon afterwards, settling in Gilmer in August, the same year. Here he soon entered upon an active practice and a promising career, but was untimely cut off by the fell destroyer, to whose rapacious demands talent, intellect, youth and promise appeal alike in vain; he is no respecter of persons. In his practice, Dr. Rainey was successful; and he devoted himself to it with an unusual assiduity and zeal. He was an honest, noble man, and a faithful friend. As a member of the Baptist church, he occupied a prominent place in the community of Gilmer, and will be sadly missed in every relation to society. The JOURNAL tenders its heartfelt sympathy to the bereaved family.

